# Glycocalyx and its involvement in clinical pathophysiologies

**DOI:** 10.1186/s40560-016-0182-z

**Published:** 2016-09-08

**Authors:** Akira Ushiyama, Hanae Kataoka, Takehiko Iijima

**Affiliations:** 1 Department of Environmental Health, National Institute of Public Health, Saitama, Japan; 2 Department of Perioperative Medicine, Division of Anesthesiology, Showa University, School of Dentistry, Tokyo, Japan

**Keywords:** Glycocalyx, Vascular permeability, Starling’s law, Endothelial surface layer, Hyaluronan, Heparan sulfate, Syndecan-1, Sepsis, Lectin, Leukocyte

## Abstract

Vascular hyperpermeability is a frequent intractable feature involved in a wide range of diseases in the intensive care unit. The glycocalyx (GCX) seemingly plays a key role to control vascular permeability. The GCX has attracted the attention of clinicians working on vascular permeability involving angiopathies, and several clinical approaches to examine the involvement of the GCX have been attempted. The GCX is a major constituent of the endothelial surface layer (ESL), which covers most of the surface of the endothelial cells and reduces the access of cellular and macromolecular components of the blood to the surface of the endothelium. It has become evident that this structure is not just a barrier for vascular permeability but contributes to various functions including signal sensing and transmission to the endothelium. Because GCX is a highly fragile and unstable layer, the image had been only obtained by conventional transmission electron microscopy. Recently, advanced microscopy techniques have enabled direct visualization of the GCX in vivo, most of which use fluorescent-labeled lectins that bind to specific disaccharide moieties of glycosaminoglycan (GAG) chains. Fluorescent-labeled solutes also enabled to demonstrate vascular leakage under the in vivo microscope. Thus, functional analysis of GCX is advancing. A biomarker of GCX degradation has been clinically applied as a marker of vascular damage caused by surgery. Fragments of the GCX, such as syndecan-1 and/or hyaluronan (HA), have been examined, and their validity is now being examined. It is expected that GCX fragments can be a reliable diagnostic or prognostic indicator in various pathological conditions. Since GCX degradation is strongly correlated with disease progression, pharmacological intervention to prevent GCX degradation has been widely considered. HA and other GAGs are candidates to repair GCX; further studies are needed to establish pharmacological intervention. Recent advancement of GCX research has demonstrated that vascular permeability is not regulated by simple Starling’s law. Biological regulation of vascular permeability by GCX opens the way to develop medical intervention to control vascular permeability in critical care patients.

## Background

More than 70 years ago, Danielli [1] and Chambers and Zweifach [2] introduced the concept of a thin non-cellular layer on the endothelial surface. This layer was thought to include absorbed plasma protein, although a direct demonstration of this layer was technically impossible at that time. About 20 years later, Copley [3] reported the endothelium–plasma interface and developed a concept in which the endothelial surface was covered by a thin molecular layer and an immobile sheet of plasma. The existence of the latter structure was identified when intravital microscopy was used to examine the hamster cheek pouch. In 1966, Luft used ruthenium red staining and electron microscopy to examine the endothelial surface [4]. Using this technique, Luft directly demonstrated the existence of an endocapillary layer that had evaded visualization using light or electron microscopy; this layer had a thickness in the range of 20 nm. Subsequent studies replicated these results and led to the concept that this layer was composed of proteoglycans (PGs) and glycosaminoglycans (GAGs) with a thickness of several tens of nanometers, as has been previously reviewed [5, 6]. Since the 1970s, the development of the intravital model for studying microcirculation has enabled several indirect and direct observations of the existence of an endothelial surface layer with a gel-like endothelial glycocalyx layer (GCX) located on the luminal surface of blood vessels [5].

### Biology of glycocalyx

#### Structure of the endothelial GCX

**Fig. 1:**
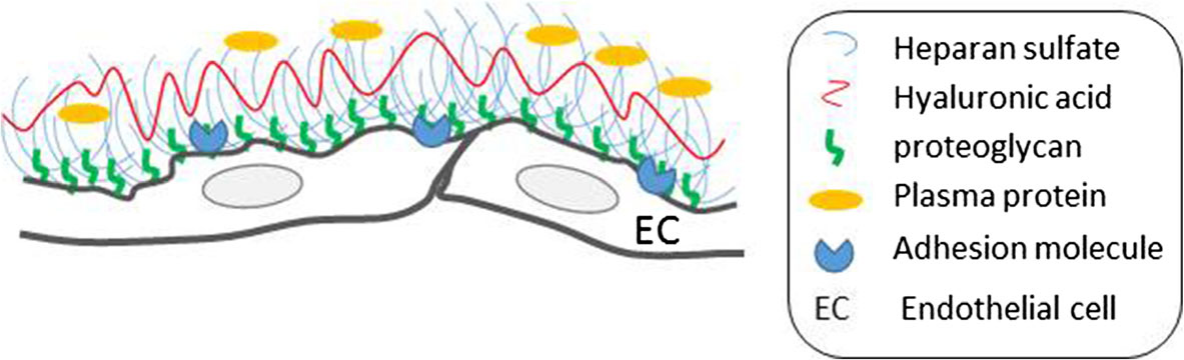
Structural diagram of the ESL. The ESL is composed of a layer of PGs and GAGs lining the luminal surface of the endothelium. The image is not shown to scale

The endothelial surface layer (ESL) is a multilayer structure that normally covers most of the surface of the endothelial cells and reduces the access of cellular and macromolecular components of the blood to the surface of the endothelium. The GCX, which is major constituent of the ESL, forms a luminal mesh that provides endothelial cells with a framework to bind plasma proteins and soluble GAGs. The GCX itself is inactive; however, once plasma constituents are bound with or immersed into the GCX, it forms the physiologically active ESL [7] (Fig. 1).

Glycoproteins and PGs form the bulk of the GCX [5, 8, 9]. PGs have a protein core to which are attached negatively charged GAG side chains. These PGs vary in the size of their core proteins, the number of GAG side chains, and their binding to the cell membrane (Table 1). The most common GAG (50–90 %) in the vascular system is heparan sulfate (HS) [10, 11], with the remainder composed of hyaluronic acid and chondroitin, dermatan, and keratan sulfates. HS is found on several core proteins including perlecan, glypican, and syndecans. Perlecan is a large HS proteoglycan found in the basement membrane. Glypicans are a family of cell surface HS proteoglycans having a glycosylphosphatidylinositol anchor [12, 13]. The syndecan family consists of transmembrane proteoglycans found in the GCX that are shed in a soluble form when the GCX becomes disordered. Each syndecan consists of an extracellular domain that contains GAG attachment sites, a single pass transmembrane domain, and a short cytoplasmic domain with phosphorylation sites. Other core proteins, such as versicans, decorins, biglycans, and mimecans, are chondroitin sulfate-bearing or dermatan sulfate-bearing proteoglycans [11, 14]. On the other, hyaluronic acid is a GAG that does not have the ability to bind to a protein core.

**Table 1:** Characterization of proteoglycan core proteins in glycocalyx

Core protein	Core size (kDa)	Number of subtype	Structure characteristic	Linked GAG
Syndecan	19–35	4	Transmembrane protein	HS, CS
Glypican	57–69	6	GPI-anchored protein	HS, CS
Perlecan	400	1	Secreted	HS, CS
Versian	370	1	Secreted	CS, DS
Decorin	40	1	Secreted	CS, DS
Biglycan	40	1	Secreted	CS, DS
Minecan	35	1	Secreted	KS

*HS* heparan sulfate, *CS* chondroitin sulfate, *DS* dermatian sulfate, *KS* keratan sulfate

The composition and dimensions of the GCX fluctuate as it continuously replaces material sheared by flowing plasma [15], while throughout the vasculature, the thickness varies tenfold from several hundreds of nanometers to several micrometers [8]. The GCX forms a luminal mesh that provides endothelial cells with a framework to bind plasma proteins and soluble GAGs [16, 17].

### Physiological function of the ESL

#### Vascular permeability barrier

The ESL and the GCX regulate vascular permeability [18]. The charged and complexed mesh structure of the GCX acts as a macromolecular sieve [16], repelling negatively charged molecules as well as white and red blood cells and platelets. For example, macromolecules larger than 70 kDa are known to be excluded from the GCX. Albumin is 67 kDa and has a net negative charge but binds tightly to the GCX [5] because of its amphoteric nature (it carries some positive charges along the protein chain). This binding reduces the hydraulic conductivity across the vascular barrier; therefore, some albumin leaks through the GCX [19]. Some pathophysiological statuses that are accompanied by the disruption of the GCX can lead to hyperpermeability.

#### Mechanotransduction

The GCX also acts as a mechanotransducer, transmitting shear stress forces to endothelial cells thorough its intracellular protein domain [8, 18]. Conformational changes in the GCX, which can be induced by blood flow, trigger the release of nitric oxide, thereby contributing to the regulation of vasomotor tone and the peripheral distribution of oxygen. The GCX thus contributes to the maintenance of homeostasis in the peripheral tissues through this rheological mechanism [20].

#### Vascular protection via the inhibition of coagulation and leukocyte adhesion

The GCX has been shown to be a significant binding site for blood proteins, such as antithrombin III, fibroblast growth factor, and extracellular superoxide dismutase. Based on these interactions, the most important physiological role of the endothelial GCX is vascular protection via the inhibition of coagulation and leucocyte adhesion [21, 22].

Cell adhesion molecules on the endothelium, such as integrins and immunoglobulins, are buried deep within the ESL. Under inflammatory conditions, the activation and/or externalization of proteases or glycosidases can lead to the degradation of the GCX through the digestion of PGs and/or GAGs. Shedding of the GCX may facilitate ligand-receptor interactions that promote the adhesion of leukocytes [23].

## Research methods

### Ultrastructure observation by electron microscopy

**Fig. 2:**
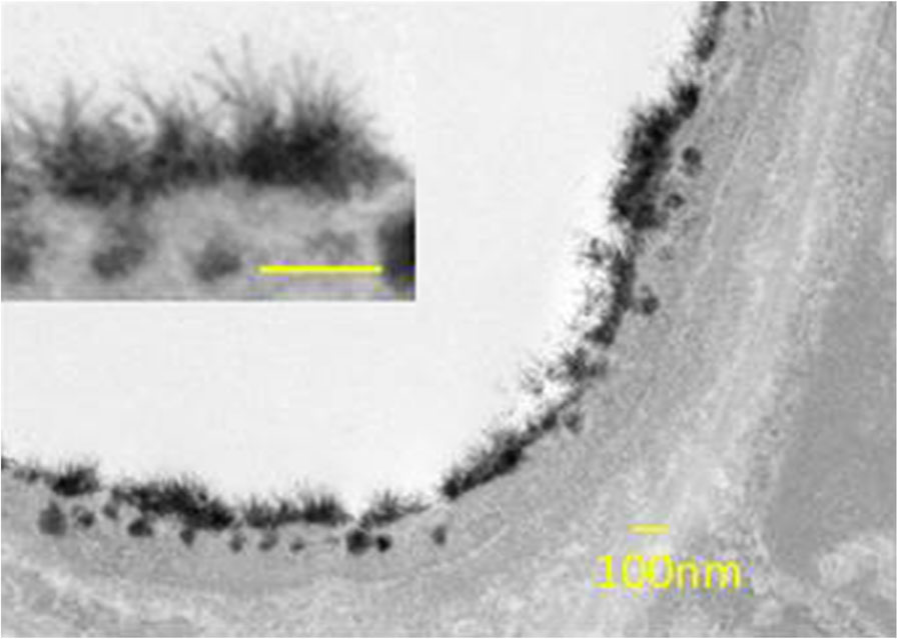
GCX layer visualized using transmission electron microscopy. Mice were fixed by perfusion with glutaraldehyde-lanthanum solution. The photos show a post-capillary venule under normal conditions. (The image was originally obtained by H. Kataoka)

The first image of the endothelial GCX was obtained using conventional transmission electron microscopy (TEM), which revealed a small layer approximately 20 nm thick in capillaries [4]. Since then, several TEM approaches, along with various perfusates or fixatives, have demonstrated stained GCX structures with large variations in thickness [16, 24]. When fixation techniques were applied to stabilize and prevent the loss of negatively charged structures, such as lanthanum [25], evidence of a thick ESL (up to approximately 800 nm in width) was obtained [26, 27]. Lanthanum clearly stains the hair-like structure of GCX, which enables to measure the thickness of the GCX (Fig. 2). The differences in GCX thicknesses and structures can likely be attributed to the use of different TEM approaches and fixation methods (perfusion or immersion). The use of alcohol during specimen processing can led to the considerable collapse of the dehydrated gel-like state of the GCX and replacement with organic solvents. To avoid shrinkage by dehydration, Ebong et al. used rapid freeze technique to preserve the native state of the GCX structure, which preserves a high water content, with which thicknesses were quantified as 6 μm for rat fat pads and 11 μm for bovine aorta [28]. The thickness of GCX may be longer than ever expected. The measurement of thickness is also largely different between visualization techniques.

### Visualization by intravital microscopy

Direct visualization of the GCX can be performed using several approaches, most of which use fluorescent-labeled lectins that bind to specific disaccharide moieties of GAG chains [29].

It has been examined a variety of fluorescent-labeled lectins for visualizing the ESL in vivo using fluorescence microscopy and shown that the specific binding of FITC (fluorescein isothiocyanate)-labeled WGA (wheat germ agglutinin) to the luminal surface of the vessel could be appropriately monitored in a mouse dorsal skinfold window [30, 31].

Recently, a novel technique that directly visualizes larger vessels using a two-photon laser scanning microscope (TPLSM) enabled a detailed description of the endothelial surface and the identification of the GCX [32, 33] because of its enhanced penetration depth, good resolution, and optical sectioning. It has been reported that thickness of the GCX of intact mouse carotid arteries was 4.5 μm by means of this technique [11].

### Functional analysis

#### Leukocyte-endothelial interactions

**Fig. 3:**
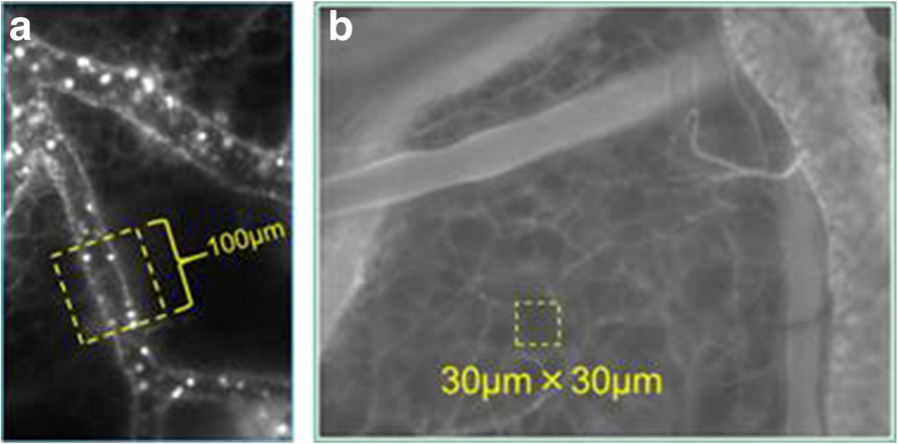
Typical experimental methods used to analyze GCX/ESL function. **a** Fluorescent-labeled leukocytes in microvasculature. To quantify the leukocyte-endothelium interaction, fluorescence-labeled leukocytes in flowing blood were observed within a region of interest (ROI) during a 30-s video recording, and adhesive and/or rolling leukocytes were counted. **b** Permeable analysis using FITC dextran. To analyze vascular permeability, fluorescence-labeled dextran was injected and time-dependent changes in brightness within an ROI (*yellow box*) set over the interstitium were identified using image analysis software. (These images were originally obtained by H. Kataoka)

Although the morphological profile of the GCX has begun to be elucidated, functional analyses are now needed to clarify the roles of the GCX. Receptors on the surface of the endothelium are assumed to hinder behind the GCX, and the degradation of the GCX exposes these receptors and triggers leukocyte-endothelial interactions. Lipopolysaccharide (LPS) may be a useful tool for triggering GCX degradation [34]. GCX degradation leads exteriorization of ICAM-1 (intercellular adhesion molecule 1) and/or VCAM-1 (vascular cell adhesion molecule 1) to the lumen of vasculature, which enhances leukocyte-endothelial interactions [35, 36]. The rolling leukocyte on the vessel wall is visualized in the septic model where the leukocyte is labeled with rhodamine 6G (Fig. 3a).

The heparanase-mediated mice also lose the ESL, which leads to the exposure of ICAM-1, VCAM-1 to circulating activated neutrophils, facilitating their adherence and extravasation [22, 37, 38]. Increases in the expressions of E-selectin, ICAM-1, and VCAM-1 have been reported in human microvascular endothelial cells [39, 40] and mice [41]. Although the importance of the GCX is being recognized, further study is needed to clarify the integrated mechanisms involved in the loss of the GCX and leukocyte-endothelium interactions.

#### Vascular permeability

Another functional role of the GCX is as a barrier to vascular permeability. To observe changes in vascular permeability in vivo, a dye extraction method, such as the Evans blue method, has been used [42]. However, with the development of fluorescent imaging, the use of dextran covalently linked to a fluorophore has become the standard technique for qualifying and quantifying vascular permeability. In some studies, FITC-labeled bovine serum albumin (BSA; molecular weight, 66 kDa) has been used to determine the vascular permeability in rodent chamber models. As a substitute for BSA, dextran, a molecular weight of 70 kDa has also been used extensively, since it has a similar molecular weight. In a study performed by Alfieri [43], they used FITC albumin, and its leakage was quantified by using the alteration of fluorescence in the ROIs (region of interests) consisted of defined squares of 900 μm^2^ (30 × 30 μm) located in three distinct interstitial areas. This technique can be applied to various weights of molecules. Kataoka and colleagues modified this method; FITC-labeled dextran (70 kDa) was injected intravenously in the mouse model, and the fluorescent intensity in ROIs (30 × 30 μm; Fig. 3b) using intravital microscopy was monitored. The data enabled the quantitative and continuous analysis of permeability under septic conditions (Kataoka et al., submitted).

## Pathophysiologies involving the GCX

### Revised Starling’s law

#### The GCX layer and its mechanism for controlling fluid movement

The GCX covers the luminal surface of the endothelium, which sieves molecules to the interstitium. The sub-GCX space in the intercellular cleft also forms a buffer space for molecules from the interstitium and intravascular spaces. This fragile and tiny structure acts as a barrier for the vessels. Studies on microvascular fluid exchange have attempted to estimate the accurate Pc (hydrostatic pressure) and π (osmotic pressure) and have revealed that the sub-GCX π is lower than the interstitial π. This means that the lower π space in the intercellular cleft insulates fluid movement along the osmotic gradient.

**Fig. 4:**
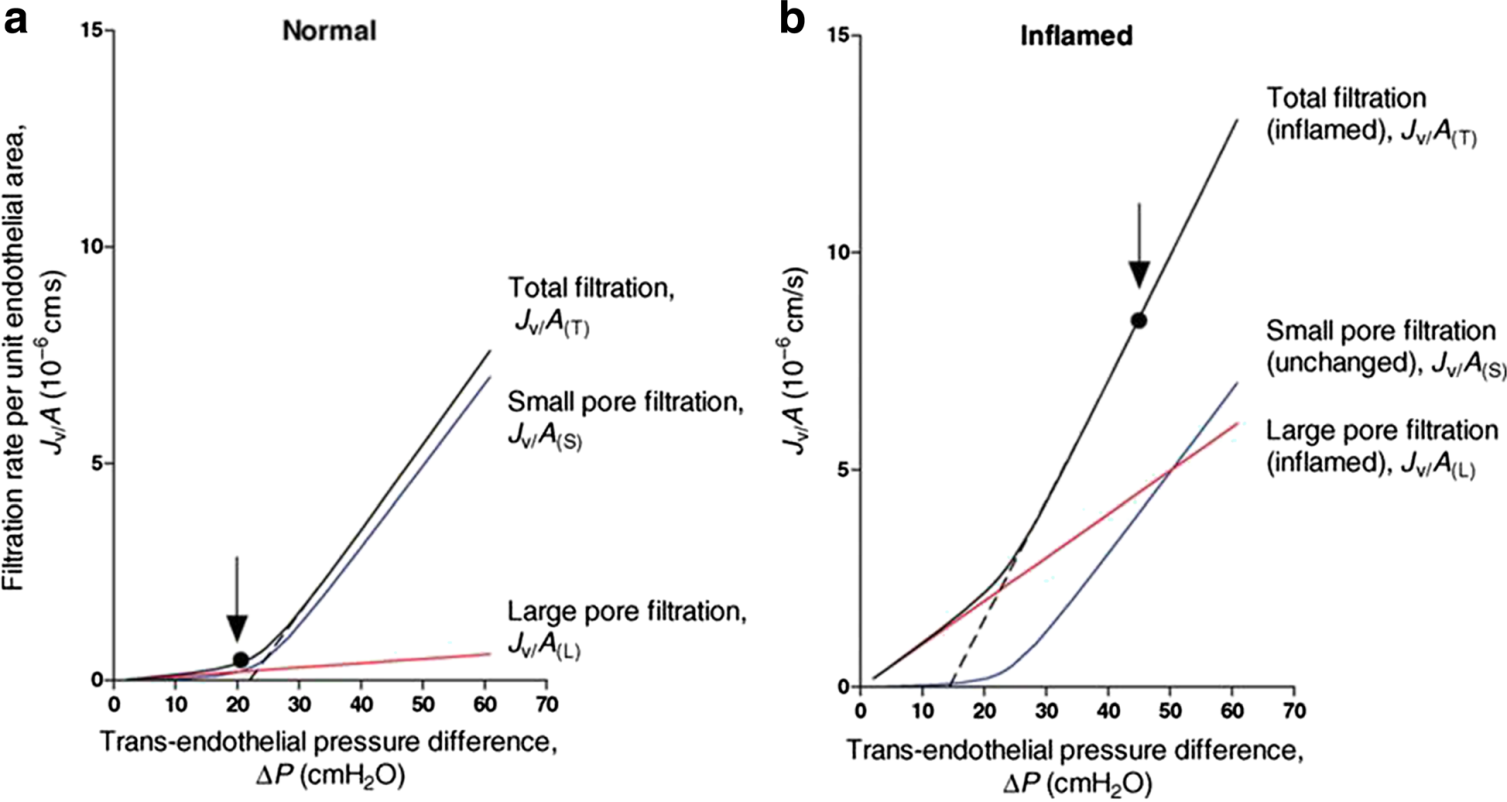
Steady-state fluid exchange simulated for a post-capillary venule, with the fluid-conducting pathways modeled as parallel small pore and large pore populations, under normal and inflamed conditions. **a** Basal low permeability state: 95 % of the hydraulic conductance is represented by small pores (radius = 4 nm; *blue curve*) and 5 % is represented by large pores (radius = 22.5 nm; *red curve*). The black solid curve shows the total fluid exchange (sum of the *red* and *blue lines*) at varying values of Pc. The vessel was perfused with Ringer solution containing serum albumin (Π*p* = 25 cmH_2_O). Pi was assumed to be constant, and the aquaporin pathway was negligible (≤10 % of total conductance). **b** Steady-state fluid exchange under increased permeability conditions in the same vessel as that shown in **a**. The *red curve* represents the flow through the large pore system after inflammation had increased the number of large pores by tenfold. The small pore population remained unchanged. The *dashed lines* represent extrapolations of the linear parts of the steady-state summed relations to the pressure axis, where their intersection gives the value of the effective COP opposing fluid filtration (reduced during inflammation). The *vertical arrows* show the typical microvascular pressures under the basal condition (*A*) and during mild inflammation (**b**). The increase in pressure contributed to the dramatic 17-fold increase in the filtration rate (cited from Levick JR, Michel CC. Cardiovasc Res. 2010;87(2):198–210.)

Based on these findings, Starling’s law for fluid movement was revised [44, 45]. According to the revised Starling’s principle, capillary hydrostatic pressure is the dominant factor in determining filtration and absorption (Fig. 4). Even at a low capillary pressure, absorption rarely occurs, and water movement is unidirectional. Under septic conditions, the profile for large pore filtration increases as the capillary pressure increases; this explains why fluid leakage is enhanced under septic conditions.

### Pathological alterations

#### GCX degradation and hyperpermeability

The GCX layer rarely allows water leakage through the ETC. However, once the GCX is disrupted, the permeability of the endothelial cells increases dramatically. Hyperpermeability induced by sepsis is a typical example in which GCX damage induces macromolecule leakage. However, the denudation of the vascular inner lumen itself cannot explain the leakage of water and other molecules, since endothelial cells bind tightly with neighboring cells via specific proteins, including cadherin and claudin [46, 47]. Therefore, the mechanism by which GCX degradation results in vascular hyperpermeability needs to be established. There are two pathways for the leakage of water and other molecules. The ETC has been suggested as one possible pathway and has been named the paracellular pathway [44]. This pathway requires the opening of intercellular keys, the proteins of which are known as tight junctions, adherent junctions, and gap junctions. This pathway seems to require intracellular signal conduction to loosen these junctions. A transcellular pathway has also been suggested. Vesicular transport to the interstitium has been confirmed during sepsis. The transcellular transport of macromolecules also results in interstitial edema.

#### GCX and vascular contraction

The GCX has been shown to sense blood flow and to regulate vascular tone via the production of NO (nitric oxide).

Yen et al. demonstrated that the denudation of the GCX by heparinase III reduced NO production; thus, the GCX has a physiological role in mechanosensing [48, 49], which may have an important role in the development of angiopathies and arteriosclerosis. According to the proposed hypothesis, GAGs holds negatively charged HS and consists of the structured water area. This area excludes the blood stream and protects the endothelial surface from being damaged. Positively charged cells or substances streaming in a column of negative charges create an electromagnetic field, resulting in the production of NO [50]. NO physiologically dilates vessels; if the dilation is sustained pathologically, NO further triggers free radicals and disrupts the ESL [51]. This disruption was suggested to trigger cholesterol accumulation, resulting in arteriosclerosis. Since the GCX is an insulator, this hypothesis is convincing. Further study may unveil the mechanism responsible for vascular aging, which would promote additional investigations of the GCX.

### Clinical implications

#### Clinical monitoring of the GCX

Angiopathy is a frequent pathological feature involved in a wide range of diseases. The GCX has attracted the attention of clinicians working on angiopathies, and several clinical approaches to examining the involvement of the GCX have been attempted. A biomarker of GCX degradation has been clinically applied as a marker of vascular damage caused by surgery. Fragments of the GCX, such as syndecan-1 and/or hyaluronan (HA), have been examined, and their validity is now being examined. Various clinical studies have also been reported.

The GCX is assumed to act as a size barrier for albumin filtration. Thus, GCX fragments could be a biomarker of renal disease [52]. Plasma HA is increased in patients with chronic kidney disease [53], kidney failure, hemodialysis, or peritoneal dialysis [54]. Whether this change should be interpreted as indicating degradation or increased turnover remains uncertain. However, a high concentration of HA seems to be a predictor of survival [55]. Acute decompensated heart failure (ADHF) is closely associated with AKI (acute kidney injury) [56]. Syndecan-1 has been assumed to be a predictor of death from ADHF [56], and syndecan-1 was selected as a significant predictor (odds ratio, 1.461; 95 % confidence interval, 1.256–1.677). In addition, biomarkers of the GCX are also being considered as possible indicators of the prognosis and diagnosis of various other diseases. Positive associations with these biomarkers have already been demonstrated for diabetes mellitus [57], cardiac surgery [58], Alzheimer disease [59], hematological disease [60, 61], and Crohn’s disease [62] (Table 2). Thus, damage to the GCX, as reflected by the plasma syndecan-1 concentration, is attracting attention in critical care fields. Even transfusions could potentially damage the GCX. Larsen demonstrated that the expression of syndecan-1 increased 24 h after red blood cell or platelet transfusion in patients with hematological disease [60]. This data suggests that the detection of GCX fragments may indicate physiological turnover of the GCX. Finally, Page et al. reviewed the clinical utility of various endothelial biomarkers for infectious disease [63] and concluded that so far, none of the examined biomarkers are clinically useful as a reliable diagnostic or prognostic indicator in sepsis.

**Table 2:** Clinical assessments of GCX damage

Fragment detection			
Authors	Subjects	Substances	Results
Ostrowski [65]	Trauma	Soluble VEGF receptor 1	Positive correlation with injury severity
Padberg [53]	CKD	Syn-1, HA, sFlt-1, sVCAM-1, vWF, angiopoietin-2	Syn-1, HA increased in parallel with CKD stage
Larsen [60]	RBC transfusion	Syn-1, slCAM-1, sVE-cadherin, hyaluronan	Slight increase in syn-1 at 24 h after transfusion
Larsen [61]	Myeloid leukemia	Syn-1, slCAM-1, sVE-cadherin, hyaluronan	High syn-1 was associated with bleeding, impaired platelet function
Neves [56]	Acute decompensated heart failure	Syn-1	Syn-1 was high in AKI
Cekic [62]	Crohn’s disease	Syn-1	Disease activity was correlated with syn-1
Page [63] (review)	Infectious disease	Ang-1,-2, vWF, thrombomodulin, sE-selectin, slCAM-1, sVCAM-1	A biomarker with consistent clinical utility was not identified
Intravital microscopy			
Authors	Subjects		Results
Nussbaum [57]	Children, diabetes mellitus type 1		GCX thickness was inversely correlated with glucose
Koning [68]	Cardiac surgery		Pulsatile and non-pulsatile reduced perfusion density zone
Broekhuizen [67]	Diabetes mellitus type 2		Sulodexide increased GCX thickness

*syn-1* syndecan-1, *HA* hyaluronan, *sFlt-1* soluble fms-like tyrosine kinase-1, *sVCAM-1* soluble vascular adhesion molecule-1, *vWF* von-Willebrand factor, *sVE-cadherin* soluble vascular endothelial cadherin

The GCX covers various receptors on the endothelial surface. Vascular endothelial growth factor (VEGF) is an important regulator of angiogenesis as well as permeability and vasodilation. This factor binds two types of receptors: VEGFR1 and VEGFR2. The binding of these receptors is regulated by soluble Fms-like tyrosine kinase receptor (sFlt-1). Reportedly, elevations in sFlt-1 are closely correlated with the APACHE II (Acute Physiology and Chronic Health Evaluation II) score, and the sFlt-1 level might be useful as a predictor of survival [64]. This receptor fragment on the endothelial surface is conceivably induced by GCX degradation. Actually, a close association has been shown between an elevation in syndecan-1 and the sVEGFR1 level (*r* = 0.76, *P* < 0.001) [65]. The appearance of this receptor fragment in the blood may reflect the extent of GCX degradation.

**Fig. 5:**
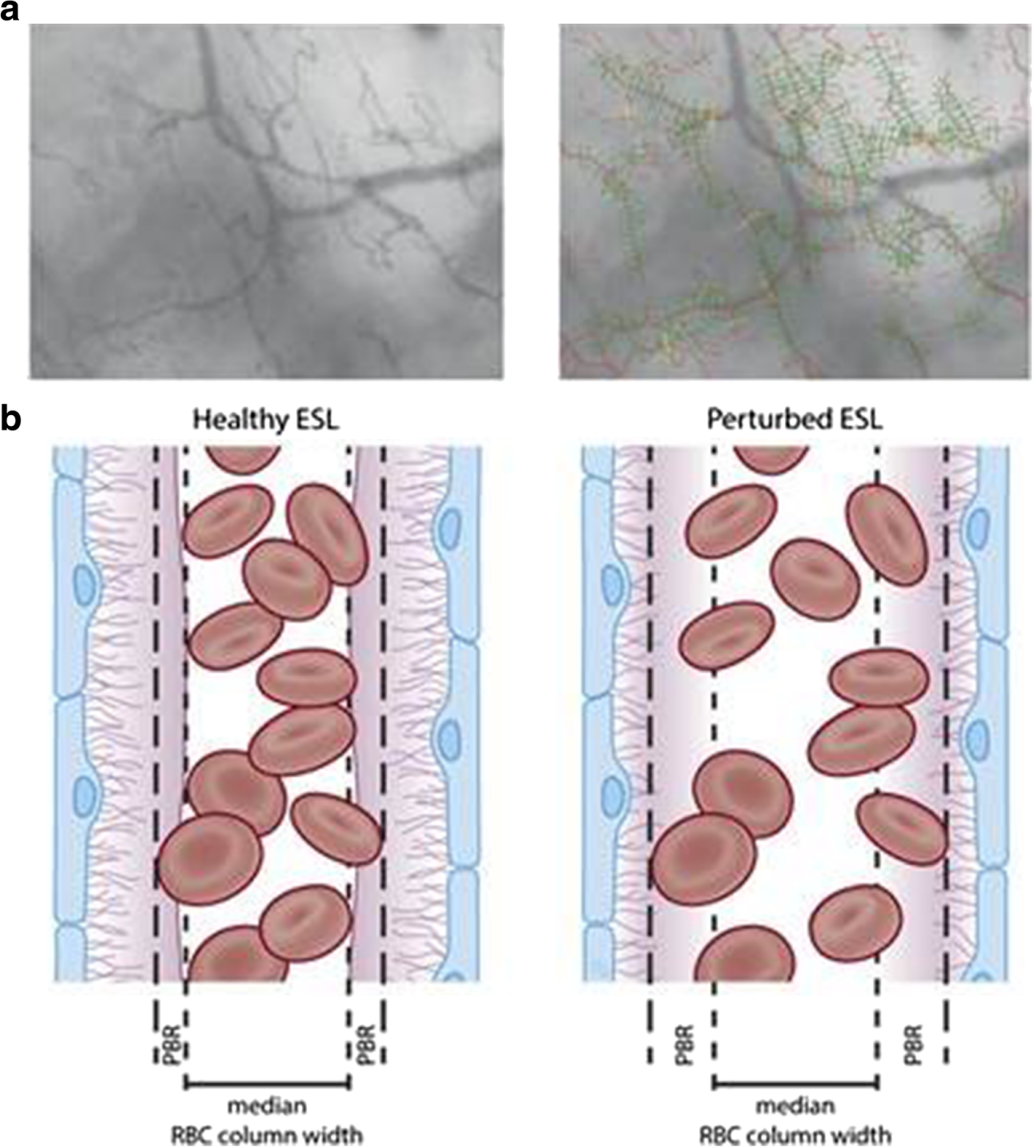
Sidestream dark field (SDF) imaging for measuring the perfused boundary region (PBR) in the sublingual capillary bed. **a** Recording of the sublingual capillary bed captured using an SDF camera (*left*). The capillaries are automatically recognized and analyzed after various quality checks (*right*). Based on the shift in the red blood cell (RBC) column width over time, the PBR can be calculated. **b** Model of a blood vessel showing the PBR under healthy conditions (*left*). The EG prevents the RBC from approaching the endothelial cell; thus, the PBR is relatively small. Under disease conditions (*right*) or after enzymatic breakdown of the EG in an animal model, the damaged EG allows the RBCs to approach the endothelium more often. This results in a higher variation in RBC column width, which is reflected as a high PBR. ESL, endothelial surface layer (cited from Dane MJ, van den Berg BM, et al. Am J Physiol Renal Physiol. 2015,308(9):F956–F966)

The diameters of peripheral vessels can be measured microscopically. The GCX layer covers the luminal surface, and red blood cells cannot pass through this layer. Consequently, visualization of the red blood cell stream can be used to demarcate the GCX layer. Several clinical studies have been reported, and changes in the GCX layer have been confirmed using this technique [66]. Sidestream dark field imaging is a unique measurement for assessing damage to the GCX in situ. This measurement observes superficial vessels (sublingual vessels) and the red blood cell stream simultaneously (Fig. 5). An exclusion space exists between the vessel wall surface and the red blood cell stream. The width of this space corresponds to the thickness of the GCX or ESL. This system can be used to estimate GCX damage in patients. Several clinical reports have already been published, and significant illness-induced changes in GCX thickness have been reported [67]. Patients who have undergone cardiopulmonary bypass (CPB) have a thinner GCX in sublingual vessels, suggesting that CPB might damage the GCX [58, 68].

#### Pharmacological preservation and intervention

Since GCX degradation is strongly correlated with disease progression, pharmacological intervention to prevent GCX degradation has been widely considered (Table 3). Hyperpermeability and thrombotic activation may be targets of such interventions. HA is expected to help repair damaged GCX [69]. Sulodexide is a highly purified mixture of GAGs composed of low molecular weight heparin (80 %) and dermatan sulfate (20 %). Sulodexide has been used to treat patients with type 2 diabetes mellitus, and a restoration of the GCX thickness was shown [67]. Antithrombin and hydrocortisone have been reported to prevent the ischemia-induced release of HA and syndecan-1 [70, 71]. Immobilizing multi-arm heparin has also been used in an animal model to prevent thrombin formation and to protect the ESL during the induction of ischemic reperfusion injury (IRI) [72].

**Table 3:** Pharmacological intervention for GCX protection

Authors	Study subjects	Substances	Insults	Results
Broekhuizen [67]	Patients	Sulodexide	Diabetes mellitus type 2	GCX thickness increased
Gao [71]	Rats	Hydrocortisone	Pancreatitis	Improved intestinal perfusion Attenuation of Syn-1 and HA release
Nordling [72]	Endothelial cells	Immobilized heparin conjugate	IRI	Attenuation of thrombotic disorder
Strunden [75]	Isolated mouse lung	HES	Heparinase	HES attenuated interstitial edema, increased pulmonary arterial pressure
Chappell [70]	Isolated guinea pig heart	Corticosteroid	IRI	Increase in Syn-1 and reduction in HS
Chappell [79]	Isolated guinea pig heart	Sevoflurane	IRI	Increase in Syn-1 and reduction in HS

*IRI* ischemia reperfusion injury

Hydroxyethyl starch has been reported to prevent capillary leakage [73], and its mechanism is assumed to have a plugging effect on ESL pores caused by GCX degradation [74, 75]. Whether the mechanism involves plugging or a specific interaction with the GCX remains uncertain [76].

Hydrocortisone is expected to reduce GCX damage [70]; this result has been obtained in an animal model, which also exhibited a reduction in sydecan-1 release, and tissue edema. Further experiments have shown that this mechanism involves the prevention of IRI-induced platelet adhesion [77, 78]. Sevoflurane also has a protective effect on the GCX by preventing IRI-induced leukocyte and platelet adhesion [79, 80].

Atrial natriuretic hormone (ANP) is assumed to cause the GCX shedding. ANP is excreted from the atrium and plays a role in regulating the intravascular volume. Physiological levels of this peptide have been shown to result in the GCX shedding and the promotion of vascular leakage [81]. Hypervolemia itself triggers ANP excretion. Since hypervolemia is harmful to thin layers, such as in the lung or other organs, excessive water should be drained. ANP may act to open water channels to the interstitium, resulting in the efflux of water [82]. Whether ANP is a regulator of the strength of the GCX seal or the disruption of the GCX is uncertain. In this context, matrix metalloprotease has been experimentally shown to reduce GCX damage. This pathway has also attracted attention in terms of protecting the GCX.

Although pharmacological intervention to GCX is widely challenged, the physiological synthesis and turn-over has not been elucidated. There may be a key point to preserve and protect GCX from various kind of injury. Albumin has been shown to reduce GCX shedding caused by cold ischemia [83]. Also fresh frozen plasma (FFP) has been shown to protect vascular endothelial permeability [84]. GCX layer is coated by albumin and proteins; thus, these natural components may not only constitute the barrier against flowing substances but may nourish GCX. Schött et al. hypothesize that FFP may inhibit or neutralize sheddases (a diverse group of proteases) and/or that FFP mobilizes intracellular stores of preformed syndecans [85]. Further research to elucidate natural turn-over of GCX may disclose the theoretical protection of GCX.

## Conclusions

The GCX is an extracellular matrix that covers the luminal surface of the vascular system. This structure is not just a barrier for vascular permeability but contributes to various functions including signal sensing and transmission to the endothelium. Thus, pathological changes to this structure are involved in the development of various diseases. Further research on the GCX is expected to provide useful information for the regulation of vascular-related pathophysiologies.

## Abbreviations

ADHF: Acute decompensated heart failure
ANP: Atrial natriuretic hormone
BSA: Bovine serum albumin
ESL: Endothelial surface layer
FFP: Fresh frozen plasma
FITC: Fluorescein isothiocyanate
GAG: Glycosaminoglycan
GCX: Glycocalyx
HA: Hyaluronan
HS: Heparan sulfate
LPS: Lipopolysaccharide
PG: Proteoglycan
TEM: Transmission electron microscopy
TPLSM: Two-photon laser scanning microscope
VEGF: Vascular endothelial growth factor
